# Supplementary material for: Effects of Si/Al Ratio on Passive NOx Adsorption Performance over Pd/Beta Zeolites
Source: Molecules. 2023 Apr 16;28(8):3501. doi: 10.3390/molecules28083501 (PMC10145102; doi:10.3390/molecules28083501)
Supplement: Supplementary file 1 [file molecules-28-03501-s001.zip › molecules-2326638-SM.pdf]

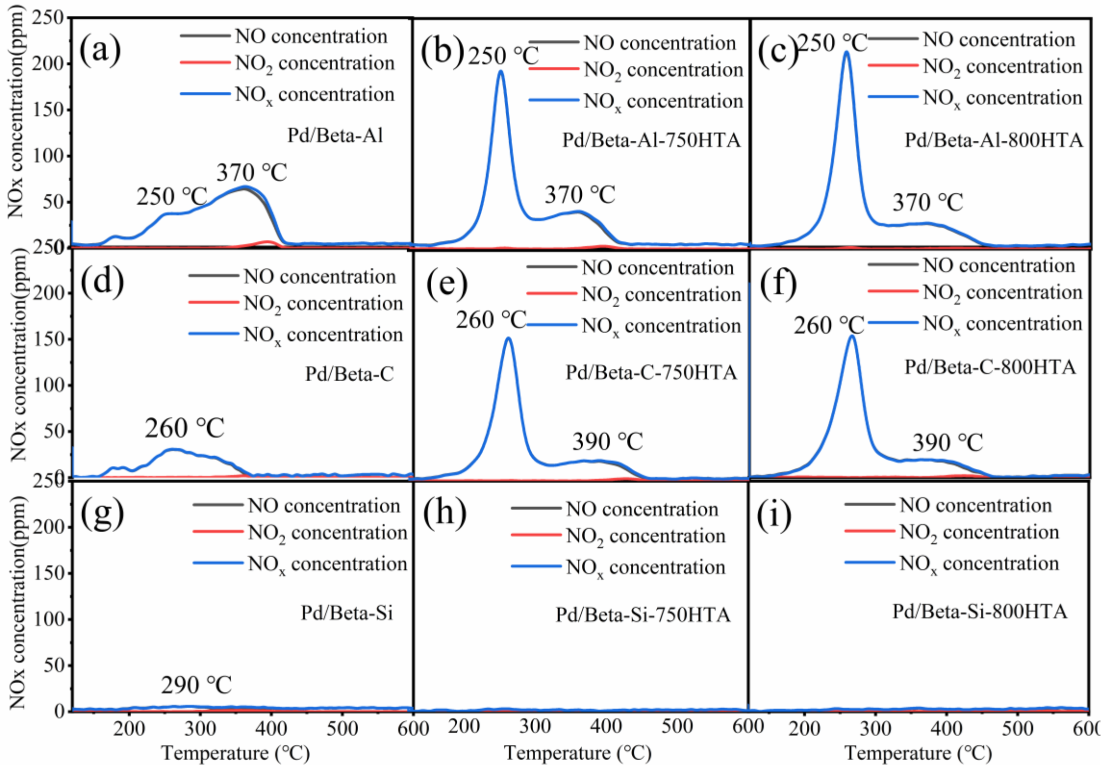

Figure S1. The NO and NO_2 storage capacity of **(a)** Pd/Beta-Al, **(b)** Pd/Beta-Al-750HTA, **(c)** Pd/Beta-Al-800 HTA, **(d)** Pd/Beta-C, **(e)** Pd/Beta-C-750HTA, **(f)** Pd/Beta-C-800 HTA, **(g)** Pd/Beta-Si, **(h)** Pd/Beta-Si-750HTA, **(i)** Pd/Beta-Si-800 HTA.

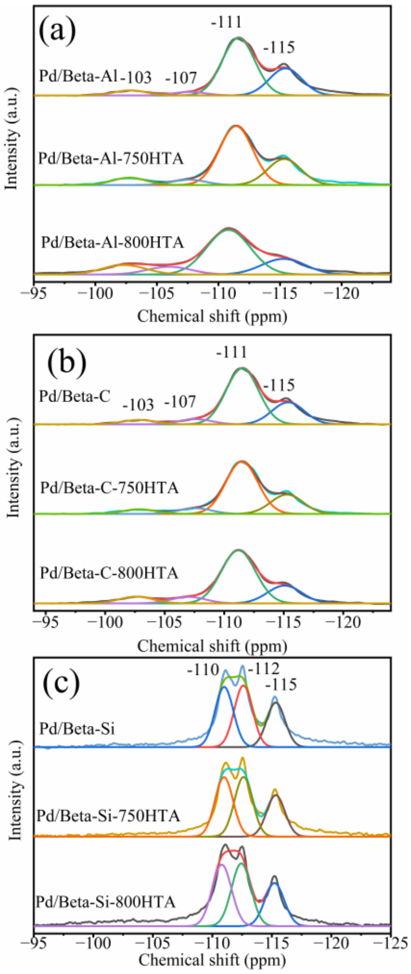

Figure S2. ^{29}Si NMR for the **(a)**Pd/Beta-Al, Pd/Beta-Al-750HTA, Pd/Beta-Al-800HTA, **(b)** Pd/Beta-C, Pd/Beta-C-750HTA, Pd/Beta-C-800HTA, **(c)** Pd/Beta-Si, Pd/Beta-Si-750HTA, Pd/Beta-Si-800HTA HTA.

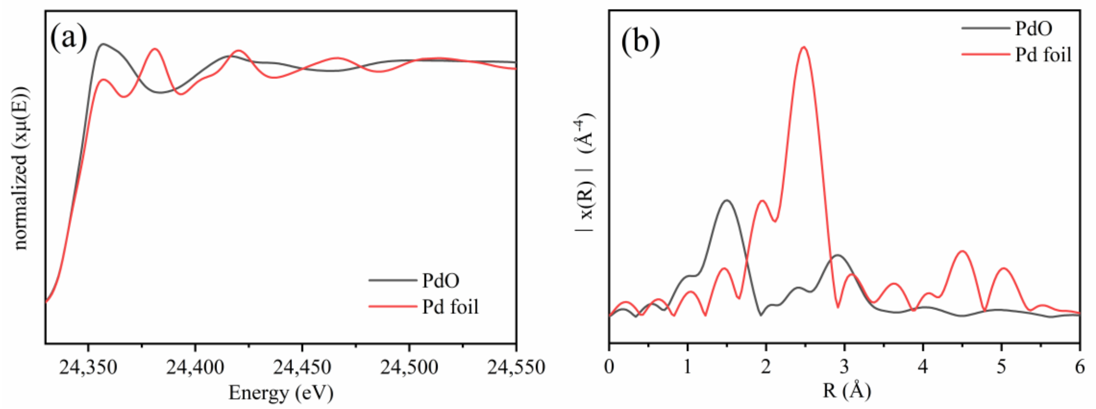

Figure S3. Pd K-edge XANES spectra **(a)** and EXAFS spectra **(b)** of PdO and Pd foil.

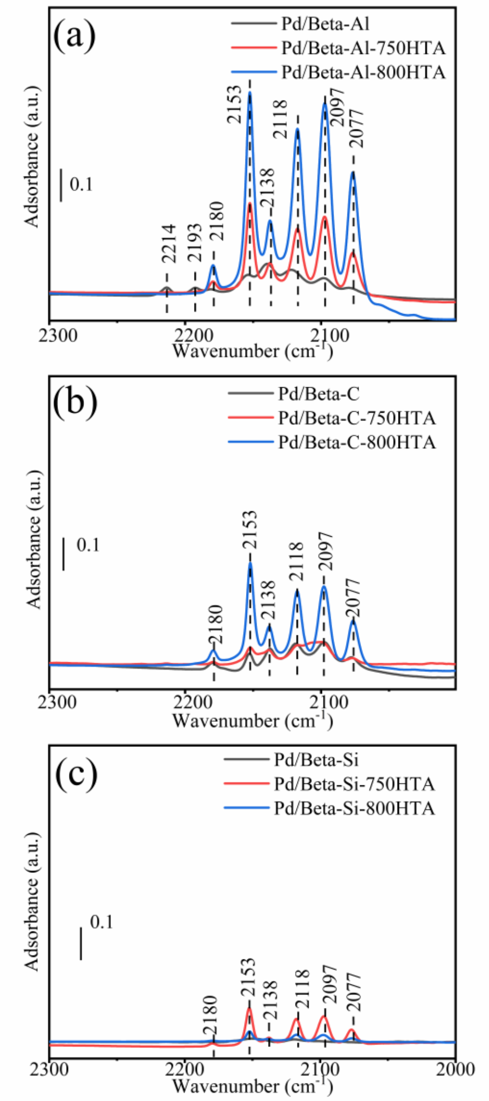

Figure S4. CO-DRIFT patterns of **(a)** Pd/Beta-Al, Pd/Beta-Al-750HTA, Pd/Beta-Al-800HTA, **(b)** Pd/Beta, Pd/Beta-750HTA, Pd/Beta-800HTA, **(c)** Pd/Beta-Si, Pd/Beta-Si-750HTA, Pd/Beta-Si-800HTA.

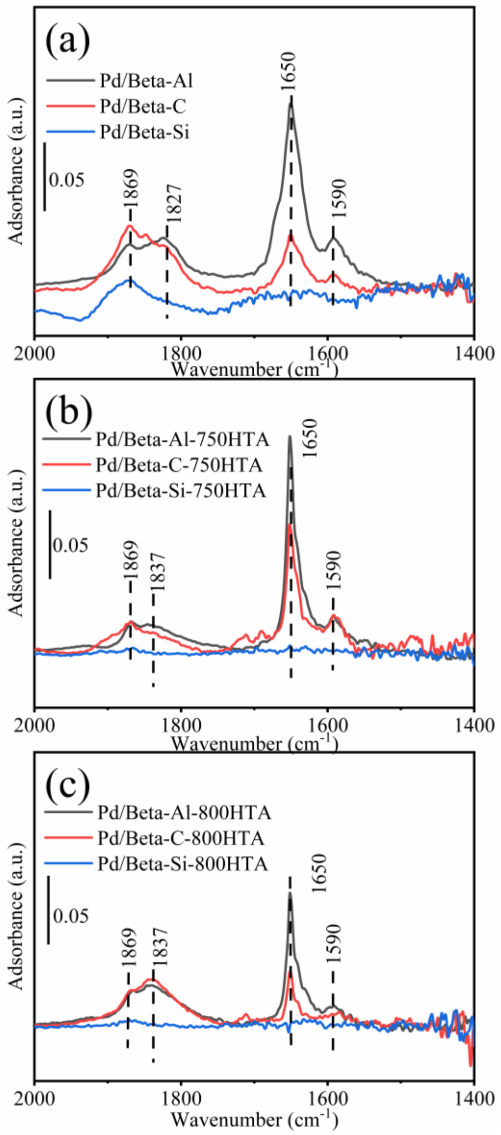

Figure S5. DRIFT spectra of **(a)** Pd/Beta-Al, Pd/Beta-C, Pd/Beta-Si, **(b)** Pd/Beta-Al-750HTA, Pd/Beta-C-750HTA, Pd/Beta-Si-750-HTA, **(c)** Pd/Beta-Al-800HTA, Pd/Beta-C-800HTA, Pd/Beta-Si-800-HTA.

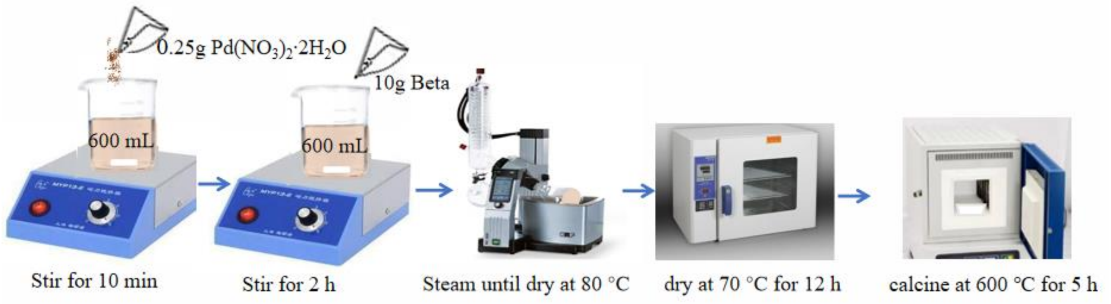

Figure S6. The synthetic path description diagram of Pd/Beta.
